# Design of Amperometric Biosensors for the Detection of Glucose Prepared by Immobilization of Glucose Oxidase on Conducting (Poly)Thiophene Films

**DOI:** 10.1155/2018/1849439

**Published:** 2018-03-01

**Authors:** Maria Pilo, Roberta Farre, Joanna Izabela Lachowicz, Elisabetta Masolo, Angelo Panzanelli, Gavino Sanna, Nina Senes, Ana Sobral, Nadia Spano

**Affiliations:** ^1^Department of Chemistry and Pharmacy, University of Sassari, 07100 Sassari, Italy; ^2^Department of Chemical and Geological Sciences, University of Cagliari, Monserrato, 09042 Cagliari, Italy

## Abstract

Enzyme-based sensors have emerged as important analytical tools with application in diverse fields, and biosensors for the detection of glucose using the enzyme glucose oxidase have been widely investigated. In this work, the preparation of biosensors by electrochemical polymerization of (poly)thiophenes, namely 2,2′-bithiophene (2,2′-BT) and 4,4′-bis(2-methyl-3-butyn-2-ol)-2,2′-bithiophene (4,4′-bBT), followed by immobilization of glucose oxidase on the films, is described. N-cyclohexyl-N′-(2-morpholinoethyl)carbodiimide metho-*p*-toluenesulfonate (CMC) was used as a condensing agent, and *p*-benzoquinone (BQ) was used as a redox mediator in solution. The glucose oxidase electrodes with films of 2,2′-BT and 4,4′-bBT were then tested for their ability in detecting glucose from synthetic and real samples (pear, apricot, and peach fruit juices).

## 1. Introduction

Qualitative and quantitative analyses in the food and beverage industry are extremely important from quality, storage, nutrition, and safety standpoints. The levels of certain sugars, like glucose, fructose, galactose, lactose, sucrose, and starch, affect intolerance conditions, diabetes, and obesity.

The determination of glucose concentration is a widespread analytical routine measurement carried out in the food industry. Glucose is often one of the most abundant monosaccharides in foods, but it is responsible for browning phenomena during dehydration and long-term storage, mainly due to the Maillard reaction. Furthermore, the accurate evaluation of the glucose amount in foods is of the utmost importance in the maintenance of its physiological level in blood of diabetics.

There are different analytical assays apt to determine glucose, colorimetric methods being the most commonly used. Today, a variety of unambiguous methods to detect and quantify glucose in assorted food matrices are available. These methods may be broadly grouped into two main categories: enzymatic approaches—including both spectrophotometric assays and glucose meters—and nonenzymatic techniques such as HPLC methods and their associated detection systems. While the former group is glucose specific, the latter is broadly adaptable and may be used to detect not only glucose but also an assortment of other carbohydrates.

In solution, glucose may exist as one of two possible anomers, termed *α* and *β*, or as the open-chain glucose aldehyde. When allowed to reach equilibrium at pH 7 and 25°C, approximately 63% of the glucose will adopt the *β*-glucopyranose conformation, with 37% existing as the *α*-glucopyranose, and less than 1% existing as either the aldehyde or as glucofuranose. Interconversion between the anomers may occur freely (albeit slowly) or via the catalytic activity of glucose mutarotases. Most enzymes preferentially bind either *α* or *β* form, but a small subset is able to utilize both anomers as the situation demands [1]. Glucose oxidase (GOx; EC 1.1.3.4) is a dimeric enzyme which catalyzes the conversion of *β*-D-glucose to D-glucono-1,5-lactone as part of the pentose phosphate pathway. The immobilization of the enzyme can be achieved using physical adsorption, cross-linking, and covalent bonding or entrapment in gels or membranes, but the main strategies employed require to entrap the enzymes into a conducting polymer film during its electrochemical growth or to attach them onto the surface of the functionalized film [2–4]. The process of entrapment involves the electrochemical polymerization of a monomer from a solution containing also the dissolved enzyme [3, 5–7]. This one-step procedure is very simple and rapid, but it has several possible drawbacks: (i) it requires that the concentration of the enzyme is fairly high, making it hardly applicable to expensive enzymes, (ii) the conditions required for the electropolymerization of the film have to be compatible with those necessary to allow the enzyme to retain its activity, and (iii) the physical entrapment within the polymer can induce a loss of the enzyme recognition or of the catalytic activity [2, 3, 7, 8]. An attractive alternative consists of a two-step approach involving, firstly, the electropolymerization of the conducting polymer on the electrode and, secondly, the chemical attachment of the biomolecules to the polymer surface [5, 9–12]. In contrast with the first procedure, this one has the advantages of allowing the use of optimal reaction conditions for each individual step, then facilitating macromolecular interactions and conserving a better access of the substrate to the immobilized biomolecule. To help to overcome the accessibility and proximity limitations and to reduce the susceptibility to interfering substances by lowering the electrode potentials, redox mediators have been used in biosensors. Mediators are small and soluble artificial electron transferring agents that can participate in the redox reaction by shuttling electrons from the redox center of the enzyme to the surface of the working electrode. Examples of commonly used mediators include quinones, organic conducting salts, dyes, ruthenium complexes, ferrocene, and ferricyanide derivatives.

Electrochemical biosensors are then a subclass of chemical sensors that use a biological recognition element (an enzyme, a protein, an antibody, etc.), which reacts selectively with the target analyte producing an electrical signal related to its concentration.

**Scheme 1:**
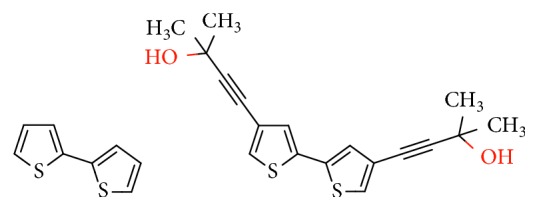
2,2′-Bithiophene (2,2′-BT) (a) and 4,4′-bis(2-methyl-3-butyn-2-ol)-2,2′-bithiophene (4,4′-bBT) (b).

Our research group is involved for several years in synthesis and characterization of thiophene-based electrochemically generated conducting polymers (CPs). In particular, our interests are concerned in terthiophenes bearing free and complexed nitrogen ligands (terpyridine or phenanthroline) [13–16]. More recently, we started to study new thiophene-based structures and their application in amperometric sensors field. In this context, we want to investigate the possibility to use electrogenerated polythiophene films in the construction of GOx-based biosensors. In particular, in the present work, we compare the behavior of two biosensors based on two different films: the first one was obtained from the commercially available 2,2′-bithiophene (2,2′-BT) and the second one from a bithiophene derivative designed and synthetized in our laboratory (4,4′-bBT), both reported in Scheme 1.

Glucose oxidase was used as a (enzymatic) recognition element, and *p*-benzoquinone as a mediator in solution.

In this paper, GOx-based biosensors obtained by immobilization of the enzyme on a poly(2,2′-BT) or a poly(4,4′-bBT) film will be named poly(2,2′-BT)/GOx and poly(4,4′-bBT)/GOx, respectively.

## 2. Experimental

### 2.1. Chemicals

All chemicals are commercially available as follows: tetraethylammonium hexafluorophosphate (TEAFP_6_) from Fluka; acetonitrile, *p*-benzoquinone (BQ), N-cyclohexyl-N′-(2-morpholinoethyl)carbodiimide metho-*p*-toluenesulfonate (CMC), and glucose oxidase (GOx) from Sigma-Aldrich; 2,2′-bithiophene (2,2′-BT) from Lancaster; glucose from Carlo Erba. The synthesis of 4,4′-bBT was performed as reported in Section 2.2; all reagents used in the synthesis were from Sigma-Aldrich. Fruit juices (pear, brand “Puertosol,” peach and apricot, brand “Valfrutta”) were used in tests with real samples in 1/100 dilution.

All electrochemical tests were performed using a CHI-650 or an Autolab PGSTAT12 potentiostat interfaced with a PC using a specific software (CHI-650 and NOVA, resp.) in a three-electrode cell equipped with the following: a platinum disk working electrode (2 mm diameter), a graphite bar counterelectrode, and an SCE reference electrode, under argon atmosphere. Before use, platinum disk was polished with alumina powder (1 and 0.3 *µ*m), placed in an ultrasonic bath and then rinsed with water and acetone.

### 2.2. Synthesis of 4,4′-bBT

A two-neck 25 mL flask equipped with a condenser, a magnetic stirrer, and an argon inlet was charged under inert atmosphere with 0.2000 g of 4,4′-dibromo-2,2′-bithiophene, 0.0053 g of [1,1′-bis(diphenylphosphino)ferrocene]dichloropalladium (Pd(dppf)Cl_2_, 6.12·10^−6^ mol), and 3% of CuI in 5 mL of diisopropylamine. Then, 3-methyl-3-butyn-2-ol (1.24·10^−3^ mol) was added, and the mixture was left under stirring at reflux and monitored by TLC on alumina (eluent petroleum ether/ethyl acetate 3/2). The reaction was left to cool to room temperature and added to 50 mL of CH_2_Cl_2_ and washed with 50 mL of saturated NaHCO_3_ solution and then with 3 × 50 mL of water. The organic phase was then treated with MgSO_4_, filtered, and the solvent was evaporated under vacuum. The residue was purified through column chromatography on alumina using petroleum ether/ethyl acetate 3/2 as eluent. Yield 75%. ^1^H NMR (CD_2_Cl_2_, ppm): *δ*H = 7.32 (s, 2H); 7.16 (s, 2H); 2.04 (s, 2H, OH); 1.57 (s, 12H, CH_3_). Elemental analysis: theoretical for C_18_H_18_O_2_S_2_: C, 65.40; H, 5.49; experimental: C, 65.28; H, 5.63.

### 2.3. Biosensor Preparation

Electrochemical polymerization of 2,2′-BT and of 4,4′-bBT was carried out in a three-electrode cell containing 0.01 M monomer solution and 0.1 M TEAFP_6_ as supporting electrolyte in 5 mL of acetonitrile, purging argon gas in the cell for 20 minutes before each experiment. Polymerization of the monomers was performed by applying a potential value equal to +1.33 V for 2,2′-BT and to +1.45 V for 4,4′-bBT until a charge of 100 mC and 12 mC was passed through the polymerization system, respectively, in order to obtain an adequate film thickness [4]. The film thickness (*d*) was evaluated according to the following equation:

(1) $$\begin{array}{c} d=\alpha Q_{\text{dep}}, \end{array}$$

where *d* = thickness in nm, *α* = experimental value in nm cm^2^/mC, and *Q*_dep_ = charge density in mC/cm^2^.

According to literature [17], an *α* value equal to 2.5 was used.

The obtained films were then neutralized by keeping them at 0 V versus SCE in a 5 mL acetonitrile solution of 0.1 M TEAFP_6_ for 60 s. Finally, the films were characterized by cyclic voltammetry in the same solution used for the neutralization, washed with distilled water, and used for enzyme immobilization. The films were immersed in 2.5 mL of distilled water containing 7.5 mg of CMC, used as a condensing agent, and 30 mg of GOx during the night in the fridge at about 4°C [4, 5]. The GOx electrodes obtained were rinsed with ultrapure water and used in the glucose-sensing experiments. After use, all biosensors have been stored in the fridge at the temperature of 4°C.

### 2.4. Glucose Sensing

The glucose sensing tests were carried out at room temperature in a cell containing 20 mL of phosphate buffer 0.1 M (pH 7) and BQ 1 mM. A constant potential of +0.40 V was applied while the solution was stirred. When the background current reached a constant value, incremental amounts of a 0.2 M *β*-D-glucose aqueous solution were injected into the cell with a time interval of about 200 s, and the current/time response was recorded.

The biosensors were then tested in the same conditions described above, adding 200 *µ*L of pure juice to the phosphate buffer-BQ aqueous solution. The glucose concentration was then estimated by the standard addition method.

## 3. Results and Discussion

### 3.1. Synthesis and Characterization of Poly(2,2′-BT) and of Poly(4,4′-bBT). Calibration of Poly(2,2′-BT)/GOx and of Poly(4,4′-bBT)/GOx Biosensors

The first step in the preparation of biosensors was the synthesis of a polythiophene film on an electrode surface.

A film of poly(2,2′-BT) was obtained by electrochemical polymerization of a 0.01 M solution of monomer. Cycling the potential between 0 and 1.40 V evidenced an increasing of the current peak, and a dark film on the working electrode surface was clearly seen, proving that the polymerization occurred.

Then, the electrode was polished with alumina, and the film used for the functionalization with the GOx was obtained applying a constant potential of +1.33 V, for the necessary time to achieve a charge of 100 mC (corresponding to a thickness of the film of about 8 *µ*m) [4, 18].

**Figure 1:**
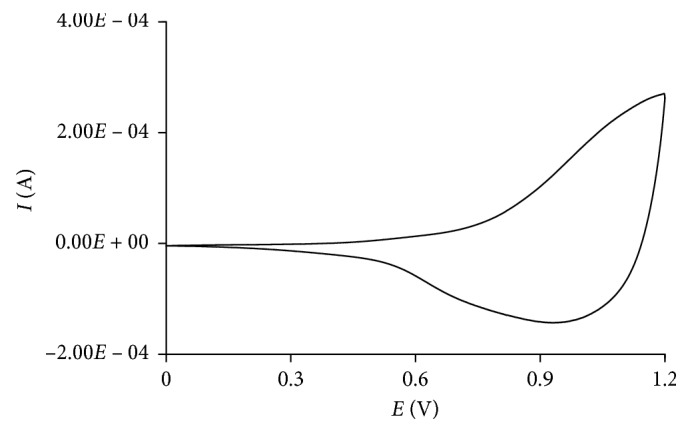
Cyclic voltammetry characterization of the poly(2,2′-BT) film in 0.1 M TEAFP_6_/acetonitrile solvent system (*Q* = 100 mC; potential scan rate = 100 mV s^−1^).

The dark red film obtained was characterized by cyclic voltammetry, evidencing a doping/dedoping response at 1.2/1.0 V, respectively (Figure 1).

The film of poly(4,4′-bBT) was deposited on the electrode surface in the same conditions as the unsubstituted polymer film. The voltammetric response in a potential range between 0 and 1.5 V evidences an increase in the peak current value at increasing scans, corresponding to the growth of a dark red film on the electrode surface.

**Figure 2:**
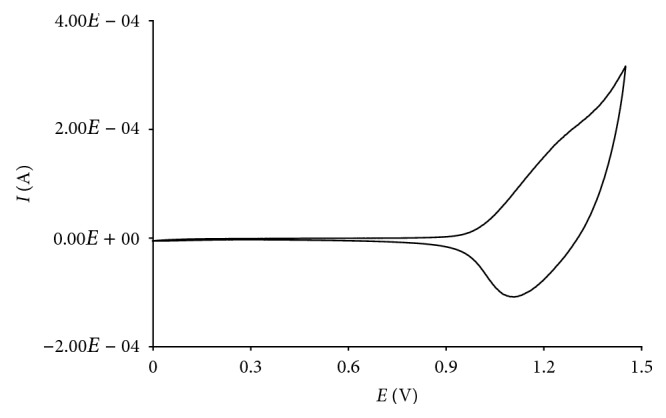
Voltammetric characterization of a poly(4,4′-bBT) film in 0.1 M TEAFP_6_/acetonitrile solvent system (*Q* = 12 mC; potential scan rate = 100 mV s^−1^).

The polymerization in potentiostatic conditions was performed applying a potential value of +1.45 V for a fixed time, corresponding to a final charge of 12 mC, and a thickness of 1 *µ*m. The characterization of the obtained film by cyclic voltammetry (Figure 2) shows a doping/dedoping process at 1.2/1.1 V, respectively.

**Figure 3:**
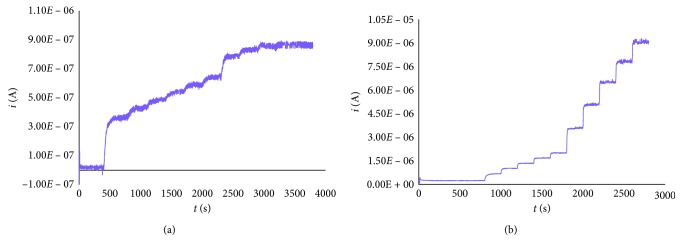
Current/time response of the biosensor poly(2,2′-BT)/GOx (a) and poly(4,4′-bBT)/GOx (b) in 0.1 M phosphate buffer (pH 7.0) and 10^−3^ M BQ with glucose concentration between 0.2 and 8.8 mM (0.2 M *β*-D-glucose aqueous standard solution).

Both the films from the unsubstituted and substituted bithiophene were modified with GOx as described in the Experimental, and the biosensors obtained were tested with regard to a current increasing corresponding to the glucose concentration increasing in the phosphate buffer solution at pH 7.0. Both devices produce, at every addition of a glucose solution, a jump-like increase of current, these being more intense at higher additions (Figure 3).

The two biosensors show a different behavior as a function of the standard additions of glucose solution: in particular, after each addition of analyte, the steady-state current has been reached much more later for the biosensor based on poly(2,2′-BT) rather than that based on poly(4,4′-bBT). This behavior likely depends also on the quite different thicknesses of the relevant polymeric films (the film of poly(2,2′-BT) is eight times more thick than that of the poly(4,4′-bBT) one).

The comparison between voltammetric characterization of poly(2,2′-BT) and poly(4,4′-bBT) shows that the two films, despite having different thicknesses, display similar current values. Such behavior suggests that the structure of the film and its conductivity depend not only on its thickness but even on the structure of the monomer. Furthermore, it can explain why two films originating from two different thiophene derivatives require a different thickness to obtain similar sensing performances of the GOx biosensors. On the other hand, the morphology of the film depends on the nucleation-growth mechanism, and in turn this mechanism is influenced by the structure of the starting species that allows obtaining a more or less dense structure [4, 13, 19]. The influence of the thickness of the films needs without doubt a more detailed study. The goal of the present paper was to investigate the possibility to use unsubstituted bithiophene and a simple, but not commercially available, bithiophene derivative in the construction of GOx biosensors. A more detailed study will be performed in the future, concerning the influence of different parameters (thickness of the film, monomer nature and concentration, and supporting electrolyte) on the behavior of the final biosensor. At the same time, a morphological characterization of different films will be carried out in order to investigate the nucleation-growth mechanism as well as to verify the porosity of the film that can tune the permeability of the film to the electrolyte, then influencing its conductivity.

In a biosensor arranged in the way described here, the conducting polymer film is at the same time the support to immobilize the enzyme and the conducting medium. The electron transfer formally occurs in the polymer film, including also the process taking place at the film-solution interface [4].

### 3.2. Validation

Validation has been accomplished for both biosensors in terms of limit of detection (LoD), limit of quantification (LoQ), linearity, response time, intermediate precision, stability, and trueness (recovery tests). LoD has been calculated by the ratio between 3.3 times the standard deviation of the blank (*n*=11) and the slope of the calibration curve obtained from a number of synthetic samples showing concentrations of the analyte very close to the expected LoD value, whereas the LoQ is measured as three times the LoD value. The intermediate precision was evaluated from data obtained from analyses of different aliquots of the same real sample (apricot juice) performed over five different analytical sessions held along two weeks and expressed as variation coefficient (CV). Stability of each biosensor was measured by performing, every two days, five consecutive measurements of concentration on a solution 1 mM in glucose. The biosensor was considered “stable” if the standard deviation of each cycle of measurements does not exceed three times the relevant intermediate precision value. Finally, trueness was estimated through recovery tests (multiple known additions of glucose on all real samples).  Table 1 gives account for all validation parameters considered here.

Validation data reveal that the poly(2,2′-BT)/GOx biosensor is characterized by a lower LoD in comparison to the poly(4,4′-bBT)/GOx one, but the remaining parameters accounted for best performances of the biosensor bearing a substituted polythiophene. In particular, the most striking differences are relative to a sharp reduction of the response time, a clear improvement of the intermediate precision and of the stability of the biosensor, and also to a quantitative recovery in the trueness evaluation.

As evidenced in Table 2, the performances of the biosensors reported in the present work are in most cases comparable to (or better than) literature data as far as linearity range, response time, and stability is concerned, in particular, in the case of polymer-type biosensors. As regards LoD, biosensors here described show values higher than literature cases. Anyway, the LoD values of poly(2,2′-BT)/GOx and poly(4,4′-bBT)/GOx are adequate for the determination of glucose in beverages like fruit juices, being a typical concentration of glucose in such matrixes between 100 and 200 mM [34, 35].

Preliminary tests on possible interfering species (ascorbic acid, uric acid, lactose, and sucrose) indicate that the combined choice of a specific enzyme as GOx and of a proper working potential (+0.40 V versus SCE) allows to make negligible the possible interferences, being the possible interfering species oxidizable at higher potential values, confirming suggestions from literature data [36].

### 3.3. Tests in Real Sample

Glucose concentration was measured in three real samples by standard addition method using both biosensors. Each juice was tested three times.

The results obtained for the mean concentration values of glucose in the three different juices using the two biosensors are reported in Table 3.

Data obtained were statistically compared by two-tailed Student's test. Difference observed between the results obtained with the two different GOx biosensors is statistically not significant for *p* < 0.05.

## 4. Conclusion

In the present paper, we report the use of polythiophene-based amperometric biosensors for the determination of glucose in aqueous solution and in real samples, namely, fruit juices. In particular, the results are focused on the comparison between a polymer film obtained from a simple bithiophene and, on the other hand, a film from a bithiophene structure leading to two unsaturated substituents on the 4 position of each heterocyclic ring. Two biosensors, poly(2,2′-BT)/GOx and poly(4,4′-bBT)/GOx, were obtained by anchoring glucose oxidase to the polymer-modified electrodes, and their behavior was tested and compared depending on the different structures of the films. According to literature references [4, 11, 12], the enzyme is reasonably assumed to be located on the surface of the polythiophene films. Being the results in aqueous solution and in real samples comparable for the poly(2,2′-BT)/GOx and poly(4,4′-bBT)/GOx, the main difference is in the different thicknesses of the film required for the optimal behavior of the biosensor. A more detailed study than that reported in this paper is required to better understand the spatial distribution of reactants and the role of polymer film thickness. Anyway, the preliminary study here reported allows doing some useful considerations. In particular, results here reported suggest that the substituted polythiophene film needs a lower thickness to obtain a biosensor performing comparably to that based on the unsubstituted film, allowing to obtain an higher stability of the film on the electrode surface and, virtually, a more effective anchoring of the enzyme and a longer life of the biosensor.

The comparison between the performances of the two biosensors allows evidencing the importance of the polymer film thickness in the behavior of the biosensor. The thickness of the film depends on the charge passed during the polymerization process. Furthermore, the thickness of the film is related to the morphology of the sensor surface that can change from a compact bilayer structure to a less dense tridimensional structure [4, 13]. The different morphologies of the film (bi- or three-dimensional) depend also on the structure of the monomer species. In this case, different tests were performed to check the more adequate thickness for both films, and the reported films are those showing the more satisfactory response concerning sensitivity, reproducibility, and stability. An adequate thickness of the film optimizes the catalytic activity of the enzyme, thus making possible a fast response of the biosensor, as well as better intermediate precision and trueness. In this context, a very important point that can influence the possibility to obtain well-performing sensors is the nature of the polymer film and its ability to give a stable adhesion to the metallic electrode surface and at the same time an efficient immobilization of the enzyme on the biosensor surface. Then, difference in the monomer units of the film can play an essential role in determining the response of the biosensor. In this paper, we explored the results deriving from an apparently simple variation on a bithiophene structure, evidencing that the proposed modification on the thiophene rings allows obtaining a more stable and more efficient sensoring device. Starting from this encouraging result, we will investigate further design able to optimize the performances of new biosensors.

## Figures and Tables

**Table 1:** Selected validation data for the determination of glucose with (a) poly(2,2′-BT)/GOx and (b) poly(4,4′-bBT)/GOx-based biosensors.

Biosensor	LoD (*μ*M)	LoQ (*μ*M)	Linearity range (mM), *Y* = *aX* + *b*; *R* ^2^	Response time (s; C glucose, mM)	Intermediate precision (CV; C glucose, mM)	Stability (days)	Trueness, recovery (% ± SD)
Poly(2,2′-BT)/GOx	30	90	0.09–5.20 *a* = 1.5 × 10^−3^ **±** 0.2 × 10^−3^ *b* = 5 × 10^−7^ **±** 6 × 10^−7^ *R* ^2^ = 0.999	180; 0.2 120; 1	7.0; 1	>15	93 ± 6

Poly(4,4′-bBT)/GOx	50	150	0.15–5.20 *a* = 3.4 × 10^−4^ **±** 0.1 × 10^−4^ *b* = 4 × 10^−8^ **±** 7 × 10^−8^ *R* ^2^ = 0.997	50; 0.2 20; 1	2.8; 1	>30	101 ± 4

**Table 2:** Comparison of the performances of biosensors proposed in the present work with literature data.

Type	Biosensor	LoD (*µ*M)	Linearity range (mM)	Response time (s; C glucose mM)	Stability (days)	Reference
Polymer	Poly(2,2′-BT)/GOx	30	0.09–5.20	120; 1	>15	*Present work*
Polymer	Poly(4,4′-bBT)/GOx	50	0.15–5.20	20; 1	>30	*Present work*
Polymer	Poly(3,4-ethylenedioxythiophene)–poly(styrene-sulfonate)/GOx		1.1–16.5	20; 1		[20]
Polymer	o-Aminophenol/GOx	0.5	0.001–1	4; 1	>300	[21]
Polymer	Poly(ethacridine)/GOx		0.01–18	2; 5	>10	[22]
Polymer	Polypyrrole/GOx		2.5–30	30; 8	>10	[23]

NM	PtNPs-MWCNTs-PANI/GOx	1.0	0.003–8.2	3; 0.6	>48	[24]
NM	AuNPs-MWCNT/GOx	2.3	0.02–10	3; 1	>7	[25]
NM	AgNPs/PANINFs/GOx	0.25	1.0–12.0	3; 1	>7	[26]
NM	PtPd-MWCNTs/GOx	0.031	0.062–14	5; 1	>7	[27]
NM	Polyelectrolyte-SWCNTs/GOx	5.0	0.5–5.0	5; 1		[28]
NM	PdNPs/CS-graphene/GOx	0.20	0.001–1.0	10; 0.08	>7	[29]
NM	Graphene nanosheet/GOx	3.0	2.0–40.0		>21	[30]

NM	Copper nanocluster/MWCNTs	0.2	0.7–3.5	5; 0.05	>35	[31]
NM	Ni nanoparticle-modified carbon paste electrode	0.3	0.001–1.0	12; 0.05	>7	[32]
NM	CuO nanowire-modified electrode	0.049	0.0004–2	1; 1	>50	[33]

NM, nanomaterials; GOx, glucose oxidase; NPs, nanoparticles; MWCNTs, multiwalled nanotubes; PANI, polyaniline; PANINFs, polyaniline nanofibers; SWCNTs, single-walled carbon nanotubes.

**Table 3:** Mean values of glucose concentration and standard deviation in three different fruit juices (pear, peach, and apricot) with two different GOx biosensors (*n*=3 replicates for each sample).

Juice	Glucose concentration (mM)		*Y* = *aX* + *b*; *R* ^2^	
	Poly(2,2′-BT)/GOx	Poly(4,4′-bBT)/GOx	Poly(2,2′-BT)/GOx	Poly(4,4′-bBT)/GOx
Pear	180 ± 20	170 ± 10	*a* = 1.4 × 10^−3^	*a* = 4.1 × 10^−4^
			*b* = 3 × 10^−6^	*b* = 6 × 10^−7^
			*R* ^2^ = 0.994	*R* ^2^ = 0.996

Peach	190 ± 10	220 ± 30	*a* = 1.1 × 10^−3^	*a* = 3.2 × 10^−4^
			*b* = 2 × 10^−6^	*b* = 5 × 10^−7^
			*R* ^2^ = 0.999	*R* ^2^ = 0.999

Apricot	240 ± 20	280 ± 50	*a* = 3.0 × 10^−4^	a = 4.3 × 10^−4^
			*b* = 3 × 10^−6^	*b* = 7 × 10^−7^
			*R* ^2^ = 0.999	*R* ^2^ = 0.999
